# Anethole as a promising antidepressant for maternal separation stress in mice by modulating oxidative stress and nitrite imbalance

**DOI:** 10.1038/s41598-024-57959-2

**Published:** 2024-04-02

**Authors:** Najmeh Rostami-Faradonbeh, Hossein Amini-Khoei, Elham Zarean, Elham Bijad, Zahra Lorigooini

**Affiliations:** 1 Student Research Committee, Shahrekord University of Medical Sciences, Shahrekord, Iran; 2 Medical Plants Research Center, Basic Health Sciences Institute, Shahrekord University of Medical Sciences, Shahrekord, Iran; 3 Department of Psychiatry, School of Medicine, Hajar Hospital, Shahrekord University of Medical Sciences, Shahrekord, Iran

**Keywords:** Anethole, Oxidative stress, Depression, Maternal separation, Mice, Natural product, Medicinal plant, Drug discovery, Neuroscience, Medical research

## Abstract

The occurrence of major depressive disorder is widespread and can be observed in individuals belonging to all societies. It has been suggested that changes in the NO pathway and heightened oxidative stress may play a role in developing this condition. Anethole is a diterpene aromatic compound found in the Umbelliferae, Apiaceae, and Schisandraceae families. It has potential pharmacological effects like antioxidant, anxiolytic, analgesic, anti-inflammatory, antidiabetic, gastroprotective, anticancer, estrogenic, and antimicrobial activities. This study aimed to investigate the potential antidepressant properties of Anethole in a mouse model experiencing maternal separation stress while also examining its impact on oxidative stress and nitrite levels. The research involved the participation of 40 male NMRI mice, separated into five distinct groups to conduct the study. The control group was administered 1 ml/kg of normal saline, while the MS groups were given normal saline and Anethole at 10, 50, and 100 mg/kg doses. The study comprised various behavioural tests, including the open field test (OFT), forced swimming test (FST), and splash test, to assess the effects of Anethole on the mice. In addition to the behavioural tests, measurements were taken to evaluate the total antioxidant capacity (TAC), malondialdehyde (MDA), and nitrite levels in the hippocampus of the mice. According to the findings, maternal separation stress (MS) led to depressive-like conduct in mice, including a rise in immobility duration during the FST and a reduction in the duration of grooming behaviour in the splash test. Additionally, the results indicated that MS correlated with an increase in the levels of MDA and nitrite and a reduction in the TAC in the hippocampus. However, the administration of Anethole resulted in an increase in grooming activity time during the splash test and a decrease in immobility time during the FST. Anethole also exhibited antioxidant characteristics, as demonstrated by its ability to lower MDA and nitrite levels while increasing the TAC in the hippocampus. The results suggest that Anethole may have an antidepressant-like impact on mice separated from their mothers, likely partly due to its antioxidant properties in the hippocampus.

## Introduction

The prevalence of depression is a significant concern in modern societies, as it can lead to a range of physical health problems^1^. An estimated 3.8% of the population experience depression, including 5% of adults (4% among men and 6% among women) and 5.7% of adults older than 60 years. Approximately 280 million people in the world have depression^2^. The depressive disorder will be the second debilitating factor until 2030^3^. Maternal care in infancy is associated with health and mental development during adolescence^4^. The separation of newborn rodents from their mothers for a specific period, known as maternal separation (MS), is an established animal model used to study the effects of stress on rodents and the development of depression-like behaviours^5^.

The maternal separation model is frequently used in animal research to examine the effects of stress and depressive behaviours on rodents^5^. The interaction between a mother and her infant is crucial for developing the brain and behaviour. Early life deficits, such as maternal separation stress in rodents, may result in altered nervous system adaptation, increasing the likelihood of experiencing depressive symptoms later in life^6^. Studies have shown that maternal separation stress during early life can negatively impact different brain regions in rodents, including the amygdala, prefrontal cortex, and hippocampus. This stress can also lead to neurotransmission alterations and affect the brain's normal functioning^7–9^. These biological, behavioural, and neurochemical changes have been linked to various neuropsychiatric conditions, including stress and depression in humans and animals during adulthood^10,11^. The use of antioxidant compounds reduces the symptoms of depression in patients. Additionally, antidepressants are able to reduce some of the markers of oxidative stress and increase some internal antioxidants^12^.

Oxidative stress is a risk element that raises oxidized lipids and proteins in the central nervous system, ultimately resulting in tissue damage^13^. Severe depression is associated with decreased antioxidants and oxidative and nitrosative activities^14^. There is considerable scientific evidence that nitric oxide (NO) may play a role in developing different neurodegenerative and inflammatory disorders^15^. Antioxidants are essential compounds that help protect cells from the harmful effects of oxidative stress, which can lead to cellular damage and contribute to the development of various diseases. Plant-based antioxidants are particularly beneficial as they contain high levels of these protective compounds. They have been shown to reduce the risk of oxidative damage in the brain and plasma, thereby reducing the likelihood of developing conditions such as inflammation, diabetes, Alzheimer's, and depression^16^.

Recent studies have focused on identifying new and effective compounds, particularly from plant-based sources, that can serve as complementary or alternative treatments for depression. These efforts are aimed at expanding the range of available treatment options and raising the quality of life with depression^17^. Anethole (1-methoxy-4-isopropinyl-benzene) is a fragrant terpenoid, colorless, sweet compound and one of the main constituents of essential oils of many plants, including anise, castor oil, and Tarragon^18,19^. Anethole has many medicinal properties, including anti-inflammatory, antioxidant, anti-neurodegenerative, and analgesic effects^20–22^.

This study aims to examine the potential antidepressant effects of Anethole in a mouse model of maternal separation stress, taking into account its potential anti-oxidative stress and anti-nitric oxide properties.

## Methods

This study is reported in accordance with ARRIVE guidelines.

### Maternal separation

The study involved pregnant NMRI mice obtained from the Pasteur laboratory and kept in conventional laboratory conditions. The maternal separation procedure, which involved separating the pups from their mothers for three hours daily from PND 2 to PND 14, was conducted as per established protocols. After PND 14, the pups were reunited with their mothers until PND 21 and were kept in groups of four in cages until PND 60. The control group, which was not subjected to any manipulation, remained with their mothers in the same cage from PND 0 to PND 21 and were then housed in groups of four in separate cages until PND 60^23^.

### Animals

The study was conducted on 40 male NMRI mice weighing 25-30g, randomly distributed into five groups. The control group (Group 1) received intraperitoneal injections of 1 ml/kg of normal saline, while the MS mice in Group 2 were administered 1 ml/kg of normal saline via the same route. The experimental groups (Groups 3, 4, and 5) consisted of MS mice that were treated with different doses of Anethole (Sigma Aldrich, St. Louis, MO, USA), which were 10, 50, and 100 mg/kg, respectively, through i.p. injections. The doses of drug administrations were chosen based on a pilot and previous studies^18,24,25^.

**Figure 1:**
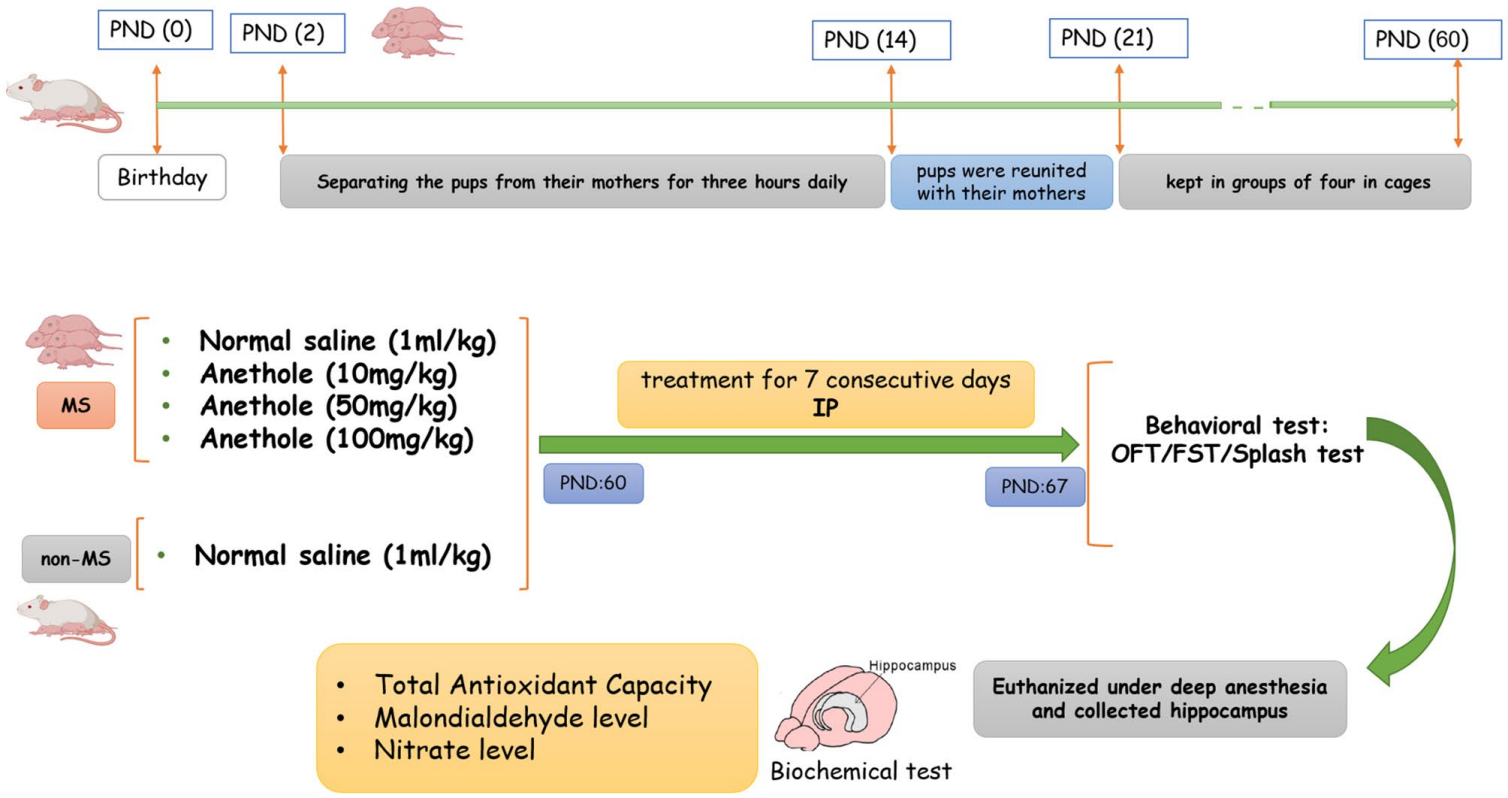
Schematic of experimental design. *PND* postnatal days, *IP* intraperitoneal injection, *FST* forced swimming test, *OFT* open field test.

The mice were treated with Anethole half an hour before the behavioural tests and continued receiving treatment for seven consecutive days. The behavioural tests, including the OFT, FST, and splash, were conducted following the treatment period. Following the completion of the behavioural tests, the mice were humanely euthanized under deep anesthesia, which was induced by administering ketamine (60 mg/kg) and xylazine (10 mg/kg) intraperitoneally. The hippocampi were collected and analyzed for antioxidant capacity, MDA, and nitrite levels (Fig. 1). The tissue was carefully extracted from the mice on a surface cooled with ice and immediately frozen in liquid nitrogen to preserve the biochemical properties of the hippocampus. Subsequently, the frozen tissue samples were transferred to a freezer at -70°C for storage until further analysis^26,27^. The α error was set at 0.05 and power (1-β) at 0.8, and the required total sample size per group was calculated as 6 in behavioral and molecular evaluations.

All methods were performed according to the relevant guideline and regulations and were approved by the Ethics Committee at Shahrekord University of Medical Sciences. The study was assigned an ethics code of IR.SKUMS.REC.1398.208. All necessary steps were taken to ensure that the welfare and ethical treatment of the mice were maintained throughout the study.

### Open field test (OFT)

OFT was used as a paradigm to test locomotion following treatment. In this study, the OFT was done immediately before the FST to consider ambulatory behavior and to confirm that adjustments in motor activity did not affect the immobility time in the FST. The open-field arena was a white plexiglass box with dimensions of 50 cm × 50 cm × 30 cm, containing 16 equal squares. Testing is conducted under dim white-light illumination (about 150 lux). The mice were arranged in the central zone of the open field, which measured 30 × 30 cm, and their movements were recorded for 5 min using a camera. Ethovision software version 8 (Noldus, Netherlands) was utilized to analyze the recorded data, and the number of horizontal and vertical movements made by the mice was accurately measured^28^.

### Splash test

The splash test was employed to evaluate the mice's self-care and motivation abilities. The assessment required applying a 10% sucrose solution on the mice's dorsal coats in their cages. Then, the duration of grooming activities was recorded for 5 min after the spraying sucrose test, which included nose/face cleaning, head washing, and body grooming. The total time spent grooming behavior was considered an index of motivation, self-care behavior, well-being, and mood. Reduced grooming behavior is considered a depression-like behavior in mice^29^.

### Forced swimming test (FST)

The FST was utilized to evaluate depression-like behaviour in mice. The FST is a commonly used method for measuring immobility in rodents. The test involved placing the mice in a cylindrical glass tank with a diameter of 10 cm and a height of 25 cm. The tank was filled with water (19 cm) at a temperature of 24 ± 1 °C. During the 6-min test period, episodes where the mice floated in the water with only necessary movements to keep their heads above water were recorded as immobility. The immobility time was recorded during the final 4 min of the test to measure the depression-like behaviour of the mice^30^.

### Measuring the antioxidant capacity of the hippocampus

The FRAP assay was used to evaluate the antioxidant power of the hippocampus. The assay measured the sample's ability to convert Fe^3+^-TPTZ to Fe^2+^. The FRAP reagent comprised TPTZ solution, FeCl3, and acetate buffer. The brain hippocampus was isolated, homogenized, and centrifuged at 10,000 rpm. A floating solution was extracted from the centrifuged sample to assess the antioxidant power. 50 μl of this solution was combined with 1.5 ml of a prepared labour solution at 37 °C. After 10 min, the formation of a complex between Fe^3+^ and TPTZ produced a blue colour that was measured for absorbance at a wavelength of 593 nm. FeSO4 was used as the standard solution for this method^31^.

### Measuring MDA of the hippocampus

The hippocampus's MDA (malondialdehyde) level was assessed by taking 1 ml of homogenized tissue and incubating it in a glass tube at 37°C for 60 min. 5% trichloroacetic acid and 67% thiobarbituric acid were added to the tube, mixed thoroughly, and centrifuged. The supernatant was transferred to another tube, heated in boiling water, and then the absorbance at 535 nm was measured to determine the MDA level in the hippocampus^32^.

### Measuring nitric oxide of the hippocampus

A colourimetric assay based on the Griess reaction was applied to assess the nitric oxide level in the hippocampus. This involved converting nitrate to nitrite using nitrate reductase enzyme and measuring the nitrite level at 570 nm with the Griess reagent. The Griess reaction produces colour from the diazotization of a sulfonamide by nitrite in an acidic environment, followed by conjugation with an aromatic amine (*N*-1-naphthyl ethylenediamine (NEDD))^33^.

### Data analysis

The obtained data were statistically analyzed using GraphPad Prism 8 software and presented as mean ± SEM. One-way ANOVA followed by Tukey's post hoc test determined significant group differences. A level of statistical significance was set at P < 0.05.

### Ethical approval

All methods were performed according to the relevant guideline and regulations.All procedures were carried out under the regulations of the University and the Guide for the Care and Use of Laboratory Animals of the National Institutes of Health (ethical code: IR.SKUMS. REC.1398.208) and Guide for the Care and Use of Laboratory Animals (8th edition, National Academies Press).

## Results

### The effect of anethole on locomotor activity in the OFT

**Figure 2:**
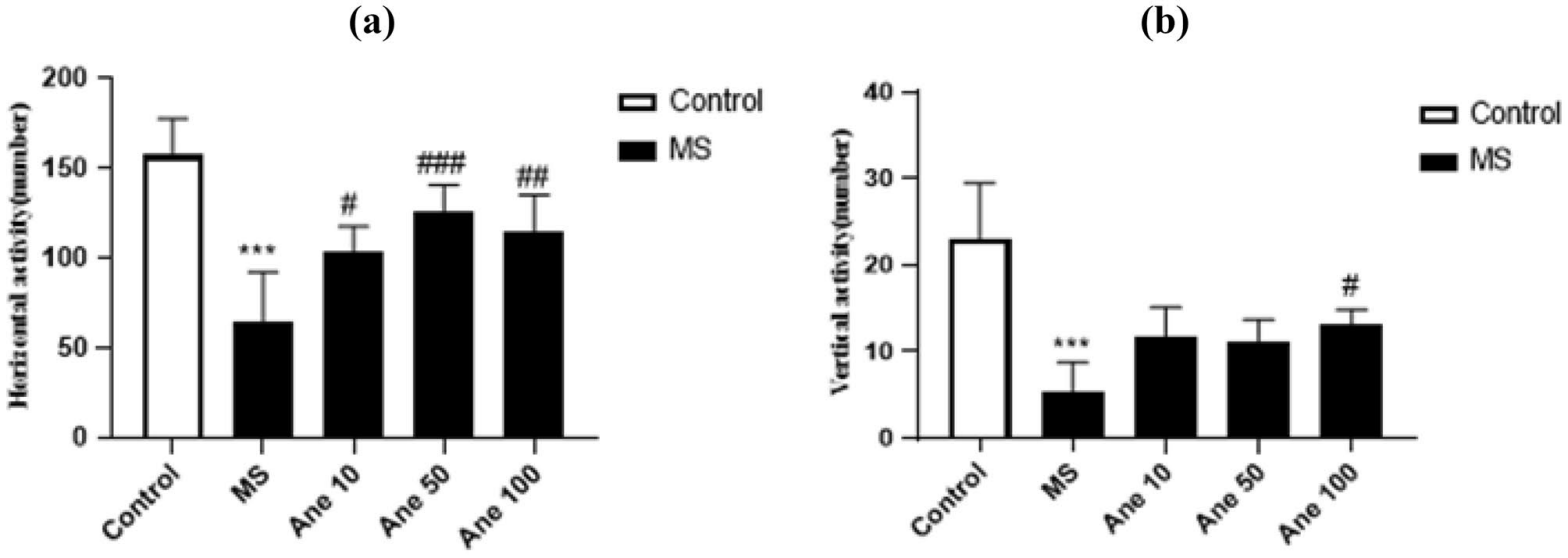
The effect of anethole on locomotor activity in the OFT. (**a**) Horizontal activity and (**b**) vertical activity. Control: group without MS receiving normal saline; MS: MS group receiving normal saline; Ane 10, Ane 50, and Ane 100: MS group treatment with anethole at doses 10, 50, and 100 mg/kg. ***P < 0.001: comparison of MS group and control group; ^#^P < 0.05, ^###^P < 0.001 and ^##^P < 0.01: comparison of MS group with MS group receiving anethole at doses 10, 50, and 100 mg/kg (**a**). ***P < 0.001: comparison of MS group and control group; ^#^P < 0.05: comparison of MS group with MS group receiving anethole at dose 100 mg/kg (**b**).

One–way ANOVA analysis showed that there are significant differences in horizontal activity (F (4, 25) = 19.96, P < 0.0001) and vertical activity(F (4, 25) = 17.75, P < 0.0001) among the experimental group. Compared to the control group, the MS group exhibited a substantial decrease in horizontal and vertical movements, with a statistically significant 0.001. However, treatment with Anethole at 10, 50, and 100 mg/kg doses lead to a considerable increase in the number of horizontal movements in the MS group (0.05, 0.001, and 0.01 levels, respectively), and a noteworthy increase in the number of vertical movements at 100 mg/kg dose (P < 0.05) (Fig. 2a, b). The observed results suggest that Anethole supplementation can improve locomotor activity in the MS group. The level of statistical significance was predetermined at P < 0.05.

### The effect of anethole on grooming activity time in the splash test

**Figure 3:**
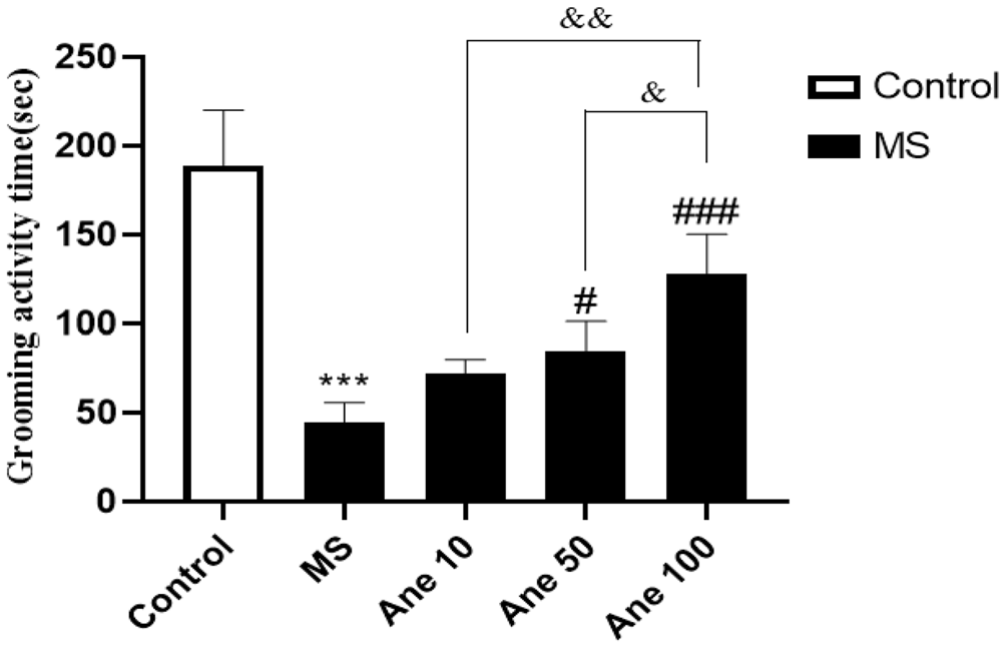
The effect of anethole on grooming time in the splash test. Control: group without MS receiving normal saline; MS: MS group receiving normal saline; Ane 10, Ane 50, Ane 100: MS group treatment with anethole at doses 10, 50, and 100 mg/kg. ***P < 0.001: comparison of MS group and control group; ^#^P < 0.05 and ^###^P < 0.001: comparison of MS group with MS group receiving anethole at doses 50 and 100 mg/kg; ^&&^P < 0.01 and ^&^P < 0.05: comparison of group receiving anethole at a dose of 10 mg/kg with the group receiving anethole at a dose of 100 mg/kg and comparison of group receiving anethole at a dose of 50 mg/kg with the group receiving anethole at a dose of 100 mg/kg.

One–way ANOVA analysis showed that there are significant differences in the grooming activity time (F (4, 25) = 60.23, P < 0.0001) among the experimental group. The findings revealed a significant reduction in grooming activity time in the MS group compared to the control group at 0.001. However, treatment with Anethole at different doses (50 and 100 mg/kg) significantly increased grooming activity time in the MS group (0.05 and 0.001 levels, respectively). Furthermore, the groups that received Anethole at doses of 10 and 100 mg/kg observed a significant effect at 0.01 level, and between the groups that received Anethole at doses of 50 and 100 mg/kg deliciated substantial differences at 0.05 in grooming activity time (Fig. 3). These results suggest that Anethole supplementation can improve grooming activity in the MS group. The statistical significance level was predetermined at P < 0.05.

### The effect of anethole on immobility time in the FST.

**Figure 4:**
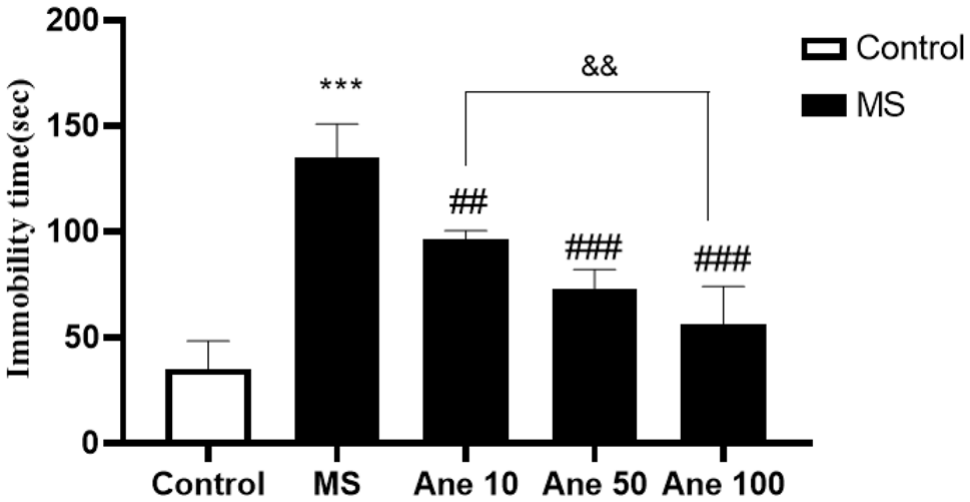
The effect of anethole on immobility time in the FST. Control: group without MS receiving normal saline; MS: MS group receiving normal saline; Ane 10, Ane 50, Ane 100: MS group treatment with anethole at doses 10, 50 and 100 mg/kg. ***P < 0.001: comparison of MS group and control group; ^##^P < 0.01, ^###^P < 0.001 and ^###^P < 0.001: comparison of MS group with MS group receiving anethole at doses 10, 50 and 100 mg/kg; ^&&^P < 0.01: comparison of group receiving anethole at a dose of 10 mg/kg with the group receiving anethole at a dose of 100 mg/kg.

One–way ANOVA analysis showed that there are significant differences in the immobility time (F (4, 25) = 63.57, P < 0.0001) among the experimental group. The analysis revealed a significant increase in immobility time in the FST for the MS group compared to the control group at 0.001. However, treatment with Anethole at doses of 10, 50, and 100 mg/kg significantly decreases immobility time in the MS group (0.01, 0.001, and 0.001 levels, respectively). Additionally, a significant difference in immobility time was observed between the group that received Anethole at doses of 10 and 100 mg/kg (P < 0.01), indicating the potential antidepressant effect of Anethole in the MS-induced depression model. Results showed that Anethole supplementation may be an effective treatment for depression. The predetermined significance level for statistical analysis was set at P < 0.05 (Fig. 4).

### The effect of anethole on antioxidant capacity

**Figure 5:**
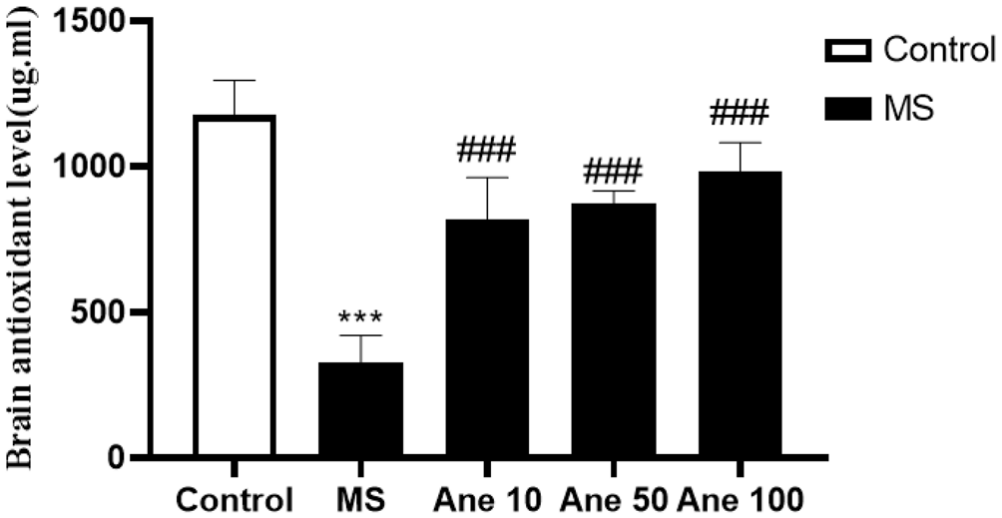
The effect of anethole on antioxidant capacity. Control: group without MS receiving normal saline; MS: MS group receiving normal saline; Ane 10, Ane 50, Ane 100: MS group treatment with anethole at doses 10, 50 and 100 mg/kg. ***P < 0.001: comparison of MS group and control group; ^###^P < 0.001: comparison of MS group with MS group receiving anethole at doses 10, 50 and 100 mg/kg.

One–way ANOVA analysis showed that there are significant differences in the antioxidant capacity (F (4, 25) = 69.92, P < 0.0001) among the experimental group. The study demonstrated a substantial reduction in the antioxidant capacity of the hippocampus in the MS group compared to the control group, with a significant difference at 0.001 level. However, treatment with Anethole at doses of 10, 50, and 100 mg/kg significantly increased hippocampus antioxidant capacity in the MS group at 0.001. Notably, no significant differences in the hippocampus antioxidant capacity were observed among the groups that received Anethole at 0.05 (Fig. 5). These findings suggest that Anethole supplementation may improve hippocampal antioxidant capacity in the MS-induced depression model. A threshold of P < 0.05 was selected as the level of statistical significance.

### The effect of anethole on MDA level

**Figure 6:**
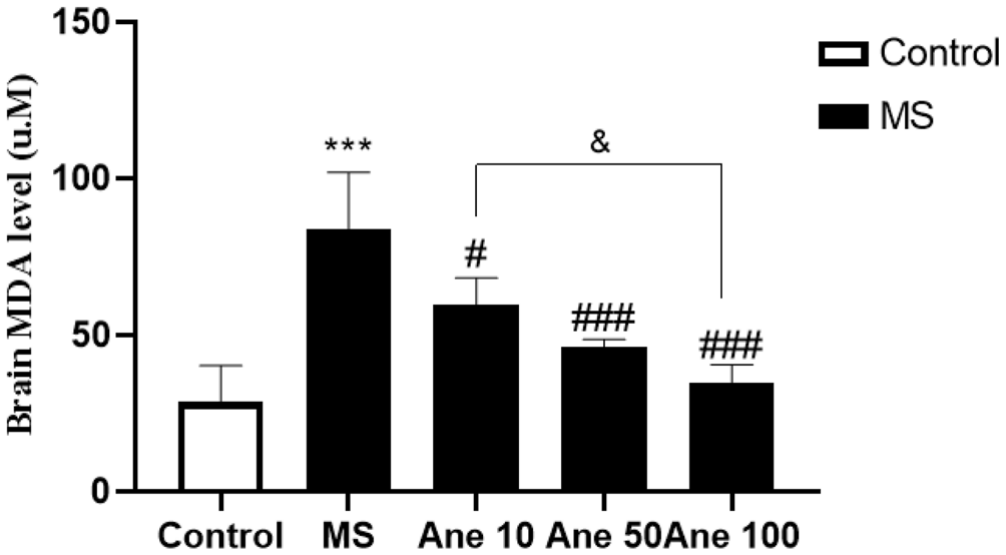
The effect of anethole on MDA level. Control: group without MS receiving normal saline; MS: MS group receiving normal saline; Ane 10, Ane 50, Ane 100: MS group treatment with anethole at doses 10, 50 and 100 mg/kg. ***P < 0.001: comparison of MS group and control group; ^#^P < 0.05,0^###^P < 0.001 and ^###^P < 0.001: comparison of MS group with MS group receiving anethole at doses 10, 50 and 100 mg/kg. ^&^P < 0.05: comparison of group receiving anethole at a dose of 10 mg/kg with the group receiving anethole at a dose of 100 mg/kg**.**

One–way ANOVA analysis showed that there are significant differences in the MDA level (F (4, 25) = 31.76, P < 0.0001) among the experimental group. The study findings revealed a significant increase in the hippocampus MDA level in the MS group compared to the control group at 0.001 level. However, treatment with Anethole at 10, 50, and 100 mg/kg doses resulted in a significant decrease in the hippocampus MDA level in the MS group (0.01, 0.001, and 0.001 levels, respectively). Additionally, a considerable difference in hippocampus MDA levels was observed between the group that received Anethole at doses of 10 and 100 mg/kg at 0.05 (Fig. 6). The study results imply that Anethole may protect the hippocampus by reducing MDA levels in the MS-induced depression model. A P-value of less than 0.05 was used as the predetermined threshold in the study to assess statistical significance.

### The effect of anethole on nitrite level

**Figure 7:**
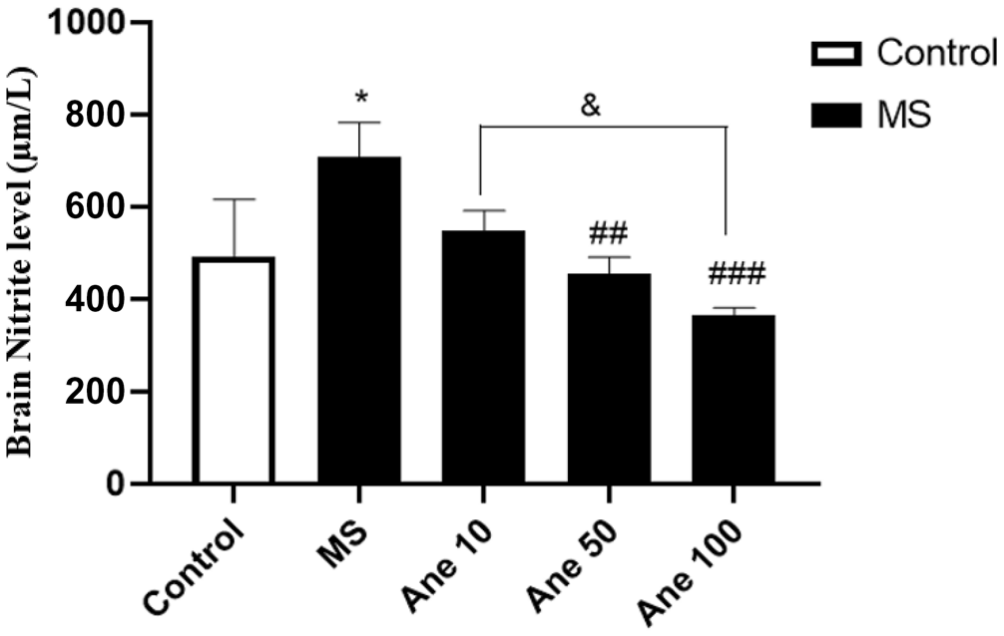
The effect of anethole on nitrite level. Control: group without MS receiving normal saline; MS: MS group receiving normal saline; Ane 10, Ane 50, Ane 100: MS group treatment with anethole at doses 10, 50 and 100 mg/kg. *P < 0.05: comparison of MS group and control group; ^##^P < 0.01 and ^###^P < 0.001: comparison of MS group with MS group receiving anethole at doses 50 and 100 mg/kg. ^&^P < 0.05: comparison of group receiving anethole at a dose of 10 mg/kg with the group receiving anethole at a dose of 100 mg/kg.

One-way ANOVA analysis showed that there are significant differences in the nitrite level (F (4, 24) = 27.43, P < 0.0001) among the experimental group. The findings indicated a noteworthy increase in the hippocampus nitrite level in the MS group compared to the control group at 0.05 level. However, treatment with Anethole at 50 and 100 mg/kg doses lead to a significant decrease in the hippocampus nitrite level in the MS group (0.01 and 0.001 levels, respectively). Additionally, a significant difference in the hippocampus nitrite level was observed between the group that received Anethole at 10 and 100 mg/kg doses at 0.05 (Fig. 7). Based on these results, it can be inferred that Anethole may have a potential neuroprotective effect against hippocampal damage induced by MS, as evidenced by the decrease in hippocampus nitrite levels. A threshold of P < 0.05 was established as the level of statistical significance.

## Discussion

Depression is a prevalent chronic illness worldwide, with increasing incidence rates. It can adversely affect all aspects of an individual's life, as well as their surroundings and loved ones^34^. There is substantial evidence to suggest that oxidative stress is a key factor in the development of depression and anxiety^35^. Potential antioxidants that hold a unique or distinctive role in treating depression include natural compounds of plant origin, especially Anethole^36^. The primary objective of this study was to evaluate the potential antidepressant effects of Anethole, a compound known for its antioxidant and other pharmacological properties, In a murine model of stress induced by maternal separation. The study aimed to examine the underlying mechanisms of Anethole's therapeutic effects, including its impact on oxidative stress and nitric oxide as a potent antioxidant agent.

In the current study, our discoveries revealed that maternal separation led to depression-like behaviours in the animal model. The open-field test results indicated that the MS group exhibited significantly fewer horizontal and vertical movements than the control group. These findings align with prior research by Amini et al. (2017), which similarly demonstrated that maternal separation during the neonatal period resulted in depressive behaviours and reduced movement in the open field test^6^. Treatment of MS groups with Anethole in OFT in all three doses caused a significant increase in horizontal activities. On the other hand, treatment of MS group with Anethole at a dose of 100 mg/kg caused a considerable rise in the number of vertical movements in comparison to MS group.

The findings from the forced swim test revealed a significant increase in immobility time in the MS group compared to the control group, which is consistent with previous research indicating a link between the MS method and the induction of depressive effects^37,38^. Furthermore, administering Anethole at all three doses to the MS group significantly reduced immobility time, with the degree of reduction depending on the specific dosage.

A notable difference in immobility time was observed between the groups that received Anethole at 10 and 100 mg/kg doses. Raman et al. (2020) aligned with our study, showing that the trans-anethole combination can reduce immobility time in FST^39^. In the splash test, a significant reduction in grooming time was observed in the MS group compared to the control group, indicating the adverse effects of maternal separation stress. These results align with a prior study conducted by Lorigooini et al. (2020), which explored the anxiolytic and antidepressant properties of trigonelline in an experimental mouse model designed to induce stress through maternal separation. The previous study also found increased immobility time in the FST, reduced movement in the OFT. It decreased grooming time in the splash test following MS induction, further supporting the negative impact of MS on behaviour^40^.

In contrast, when the MS group was treated with Anethole at doses of 50 and 100 mg/kg, a significant increase in grooming time was observed, and the degree of increase was dose-dependent. Furthermore, there were significant differences in grooming time between the groups that received Anethole at doses of 10 and 100 mg/kg and between the groups that received Anethole at doses of 50 and 100 mg/kg. The study's behavioural tests also indicated that intraperitoneal injection of Anethole resulted in a significant increase in the number of movements in the OFT, the amount of grooming in the splash test, and a decrease in immobility time in the FST when compared to the MS group. These findings suggest that Anethole may have antidepressant-like effects on behavioural tests, with the most significant impact observed at 100 mg/kg.

In oxidative stress, rising free radicals damage the nervous system in various ways, creating complications such as depression, diabetes, and Parkinson^41^. The present study results showed that the antioxidant capacity of the brain hippocampus was significantly reduced in the MS group compared to the control group.

Treatment of MS groups in all three doses with Anethole resulted in a substantial elevation of brain antioxidant capacity hippocampus. Some studies have shown that the total serum antioxidant capacity of people with significant depression decreases compared to ordinary people^14^. In the chronic stress model caused by MS, depression-like behaviours have been observed with increased corticosterone levels, decreased ACTH, and reduced serum antioxidant capacity^42,43^. Cavalcanti et al. (2012) showed that Anethole had significant antioxidant and antimicrobial properties in improving skin wound conditions^44^. According to a study conducted by Ryu et al. (2014), Anethole has been found to possess neuroprotective properties in reducing neuronal cell death due to oxygen deprivation. The compound's mechanism of action involves acting as an antioxidant and an anti-stimulant, serving as a mitochondrial protector^45^. These studies' results align with the present study, which seems that anethole combination with antioxidant properties as free radical scavengers can show antidepressant effects.

Malondialdehyde (MDA) is an essential cellular marker that indicates the level of oxidative stress and antioxidant status, representing the end product of unsaturated fatty acid peroxidation^46^. The study observed a significant increase in hippocampal MDA levels in the MS group compared to the control group, highlighting the damaging impact of maternal separation stress on the brain. However, administering Anethole at all three doses significantly reduced hippocampal MDA levels, indicating the potent antioxidant properties of the compound. Additionally, the study found a significant difference in the hippocampal MDA levels between the groups that received Anethole at 10 and 100 mg/kg doses, further underlining the dose-dependent effects of the compound. These findings suggest that Anethole supplementation could potentially alleviate oxidative stress in the hippocampus induced by maternal separation stress, thus providing a promising therapeutic strategy for depression and related disorders.

A study examining the anethole activity on pancreatic cancer in the albino mice model of the tumour showed that Anethole caused survival time increase, weight, and tumour volume decrease. Also, this combination decreases MDA and increases glutathione^47^. Previous research has indicated that Anethole's antioxidant properties are primarily due to its ability to scavenge free radicals, which can lead to elevated intracellular levels of glutathione and glutathione transferase. The compound can also effectively inhibit lipid peroxidation, further highlighting its potent antioxidant effects.^48^. These results indicate that the Anethole has effectively reduced brain hippocampus MDA.

A decrease in nitric oxide activity in the individual leads to severe depression, characterized by a measurable increase in serum nitrite/nitrate^49^. The present study results showed that nitric oxide amount in the brain hippocampus significantly increased in MS group compared to the control group. Treatment of MS groups with Anethole at 50 and 100 mg/kg doses reduced considerably nitric oxide amount in the brain hippocampus. There was a significant difference in the nitric oxide level of the brain hippocampus between the groups receiving Anethole at doses of 100 and 10 mg/kg. Wang et al. (2008) showed that inhibition of stimulating nitric oxide prevents chronic depression-like behaviour caused by stress^50^.

Domiciano et al. (2012) showed that Anethole inhibited the production or release of inflammatory mediators such as nitric oxide, prostaglandins, IL-1, TNF, and IL-17^51^. Therefore, Anethole's significantly reducing the brain hippocampus nitric oxide improved depressive behaviours. Whereas in the present study, Anethole has shown antidepressant effects, probably observed antidepressant activity of the Anethole in our study may be related to its anti-inflammatory, antioxidant, and oxidative-nitrosative stress-relief effects. Akçan et al. (2018) showed that Anethole with LD_**50**_ 1820–5000 mg/kg has no chronic toxicity^52^. The doses used in the present study are much lower than LD_**50**_ reported, which seems like other clinical studies performed on anethole^36,39,44,51^; this compound can be a good option for designing a formulation as a supplement or drug in depression in future studies.

## Conclusion

In conclusion, this study delved into the potential antidepressant effects of Anethole, a compound known for its antioxidant properties, particularly in a murine model of stress induced by maternal separation. The behavioral tests, including the open-field test, forced swim test, and splash test, consistently demonstrated that Anethole administration significantly alleviated depression-like behaviors induced by maternal separation, with dose-dependent effects. Moreover, the study unveiled Anethole's significant antioxidant properties, as evidenced by its capacity to elevate the brain hippocampus antioxidant levels and reduce malondialdehyde (MDA), a marker of oxidative stress. The observed reduction in nitric oxide levels in the brain hippocampus further suggests the potential of Anethole in relieving oxidative-nitrosative stress associated with depression. These findings not only underscore the antidepressant potential of Anethole but also highlight its broader therapeutic implications in addressing oxidative stress-related disorders, offering a promising avenue for future research and potential formulations as supplements or drugs in depression treatment.

## Data Availability

At the Medical Plants Research Center, Shahrekord University of Medical Sciences, data concerning the current study can be obtained. Correspondence and requests for materials should be addressed to Zahra Lorigooini.

## Abbreviations

NO: Nitric oxide
MS: Maternal separation
PND: Postnatal day
OFT: Open field test
FST: Forced swimming test
Fe3 + -TPTZ: Ferric-tripiridyltriazine
TBA: Thiobarbituric acid
NEDD: *N*-1-naphthyl ethylenediamine
